# Exposure to Enriched Environment Decreases Neurobehavioral Deficits Induced by Neonatal Glutamate Toxicity

**DOI:** 10.3390/ijms140919054

**Published:** 2013-09-16

**Authors:** Gabor Horvath, Dora Reglodi, Gyongyver Vadasz, Jozsef Farkas, Peter Kiss

**Affiliations:** Department of Anatomy, PTE-MTA Lendulet PACAP Research Team, University of Pecs, Pécs 7624, Hungary; E-Mails: gabor.horvathmd@gmail.com (G.H.); vadaszgyongyi@gmail.com (G.V.); jozsef.farkas@aok.pte.hu (J.F.); peter.kiss@aok.pte.hu (P.K.)

**Keywords:** MSG, neurotoxicity, enriched environment, reflex development, motor coordination

## Abstract

Environmental enrichment is a popular strategy to enhance motor and cognitive performance and to counteract the effects of various harmful stimuli. The protective effects of enriched environment have been shown in traumatic, ischemic and toxic nervous system lesions. Monosodium glutamate (MSG) is a commonly used taste enhancer causing excitotoxic effects when given in newborn animals. We have previously demonstrated that MSG leads to a delay in neurobehavioral development, as shown by the delayed appearance of neurological reflexes and maturation of motor coordination. In the present study we aimed at investigating whether environmental enrichment is able to decrease the neurobehavioral delay caused by neonatal MSG treatment. Newborn pups were treated with MSG subcutaneously on postnatal days 1, 5 and 9. For environmental enrichment, we placed rats in larger cages, supplemented with different toys that were altered daily. Normal control and enriched control rats received saline treatment only. Physical parameters such as weight, day of eye opening, incisor eruption and ear unfolding were recorded. Animals were observed for appearance of reflexes such as negative geotaxis, righting reflexes, fore- and hindlimb grasp, fore- and hindlimb placing, sensory reflexes and gait. In cases of negative geotaxis, surface righting and gait, the time to perform the reflex was also recorded daily. For examining motor coordination, we performed grid walking, footfault, rope suspension, rota-rod, inclined board and walk initiation tests. We found that enriched environment alone did not lead to marked alterations in the course of development. On the other hand, MSG treatment caused a slight delay in reflex development and a pronounced delay in weight gain and motor coordination maturation. This delay in most signs and tests could be reversed by enriched environment: MSG-treated pups kept under enriched conditions showed no weight retardation, no reflex delay in some signs and performed better in most coordination tests. These results show that environmental enrichment is able to decrease the neurobehavioral delay caused by neonatal excitotoxicity.

## 1. Introduction

The first description of the positive effects of environmental enrichment comes from the neuroscientist, Donald P. Hebb. In his paper in 1947, he described that the animals kept as pets, *i.e.*, under enriched environment, showed better performance in memory and learning tasks [1]. Since then, hundreds of experimental data have accumulated regarding environmental factors and their importance. Among others, enriched environment has been shown to influence the development of the nervous system including that of the visual system [2–4]. It is proven that environmental factors have a major influence on the outcome of different neuronal lesions [5] and so environmental enrichment is a popular strategy to counteract central nervous system injuries. Recently we have shown, for the first time, that environmental enrichment has a protective effect in neonatal lesion of the retina [6,7]. Numerous studies have proven that enriched environment can reduce lesions induced by toxic [8–10], ischemic [11–13] and traumatic [14] injuries. The mechanism underlying this protective effect includes stimulating synaptic plasticity and neurogenesis, increase of dendritic spines and stimulating the expression of neurotrophic factors like brain-derived neurotrophic factor, insulin-like growth factor and nerve growth factor [2,15,16].

Several neurotoxicants are known to induce neurodegeneration and have negative behavioral consequences in the developing or adult brain. Among toxic lesions, lead-induced injury has been proven to be counteracted by environmental enrichment [9,17]. In our model we used monosodium-glutamate (MSG). This agent is also known as umami or E621 additive food flavoring agent. MSG has toxic effects only if it reaches the central nervous system in high concentration, which happens only in the newborn rodent brain, when MSG can pass through the blood-brain barrier. Glutamate toxicity occurs through overexcitation of the N-methyl-D-aspartate **(**NMDA) receptors. This then leads to increased calcium ion influx and finally cell death. MSG toxicity in rodents is known to cause degeneration of the retina, optic nerve, arcuate nucleus and various parts of the cortex [18–22].

The development of newborn rats during the first three postnatal weeks follows a general pattern of reflex appearance and maturation of motor skills. Previously we have shown how different perinatal insults delay neurobehavioral development: most severe retardation was observed in hypoxia and asphyxia, while perinatal stress only led to minor changes [20,23–25]. We have demonstrated that treating newborn rats with MSG delays the development of neurological reflexes and motor coordination skills [20,21].

The aim of our study was to investigate whether environmental enrichment alters neurobehavioral development and whether it provides protection against the neurobehavioral consequences of neonatal MSG toxicity.

## 2. Results and Discussion

### 2.1. Somatic Development

**Figure 1:**
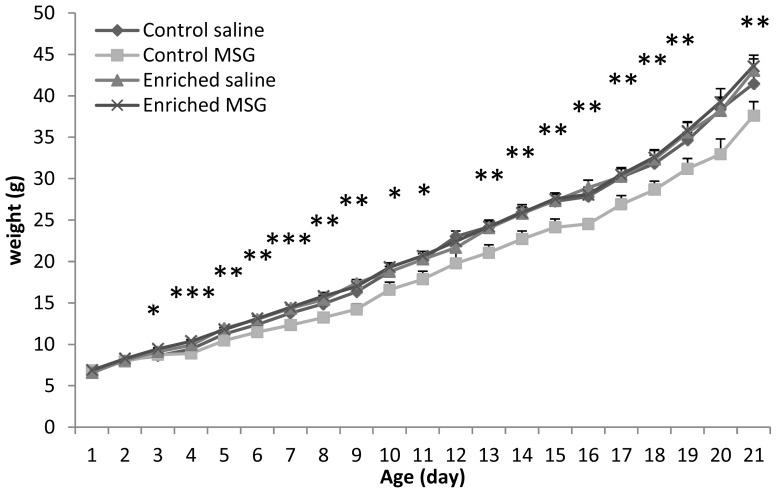
Daily changes in body weight (*: *p* < 0.05, **: *p* < 0.01, ***: *p* < 0.001 control monosodium glutamate (MSG) *vs.* all other groups).

As we have previously described [20], control animals undergoing neonatal MSG treatment had significantly less weight-gain during the entire observation period (Figure 1). However, there was no difference in the body weight between saline and MSG-treated pups kept in enriched environment. Enriched environment alone did not lead to increased weight gain in saline-treated animals. These data clearly show that enriched environment can prevent the reduced weight gain in a neonatal excitotoxic lesion.

### 2.2. Physical Parameters and Reflex Development

**Figure 2:**
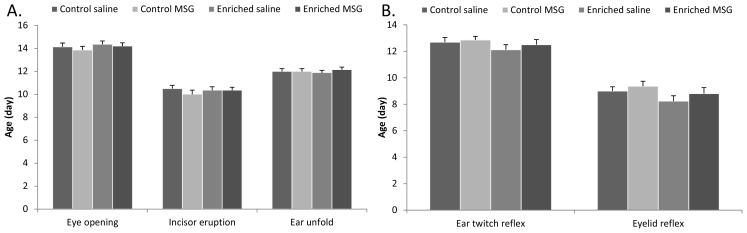
(**A**) Development of physical parameters: Eye opening, incisor eruption and ear unfold; (**B**) Ear twitch reflex and eyelid reflex.

There was no significant difference between control saline and control MSG-treated groups in the appearance of physical parameters such as eye opening, incisor eruption and ear unfolding (Figure 2A). This is in accordance with our previous observations [20]. Furthermore, this pattern was not altered by enriched environment (Figure 2A). Similarly, there was no difference between the appearance of ear twitch and eyelid reflexes (Figure 2B).

**Figure 3:**
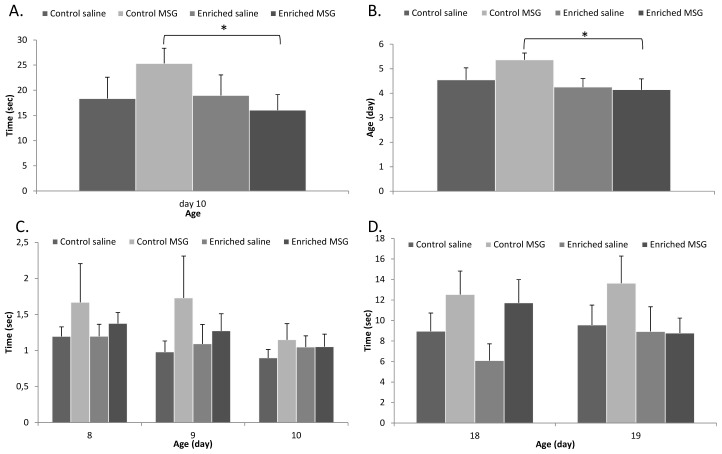
Development of reflex performance. (**A**) Gait performance; (**B**) Air righting reflex appearance; (**C**) Surface righting reflex performance; (**D**) Negative geotaxis performance (*: *p* < 0.05).

Among the other neurological reflexes, slight, but not significant, delays were observed in the day of the appearance in almost all reflexes in the MSG-treated control animals. In contrast, most signs appeared earlier in animals kept in enriched environment, but differences were not significant (data not shown). The time to perform certain reflexes (gait, air righting and surface righting reflexes) was also measured and we found no differences in performance time between control and enriched saline-treated animals. However, MSG-treated control rats took significantly longer times to perform the same task and this difference between MSG- and saline-treated animals disappeared under enriched environment (Figure 3A,B). In case of negative geotaxis and surface righting, we observed a difference, although not significant, between saline- and MSG-treated animals in both control and enriched groups (*i.e*., MSG-treated animals showed worse performance in negative geotaxis, Figure 3C,D). However, this difference disappeared later in the enriched group (Figure 3C,D).

### 2.3. Motor Coordination

**Figure 4:**
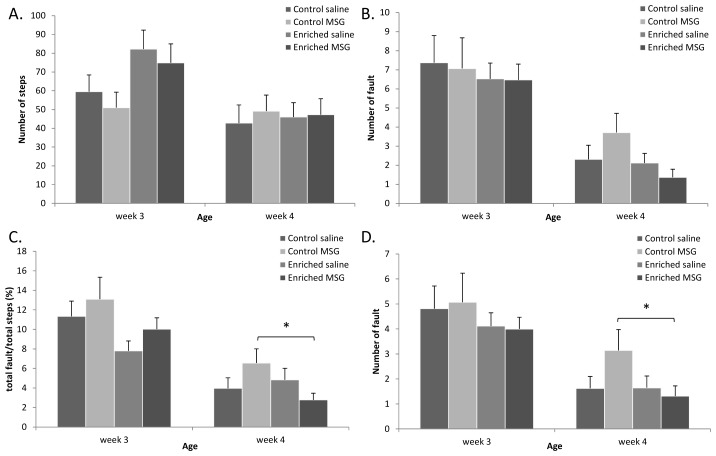
Motor coordination tests I: Grid walking and footfault test. (**A**) Number of total steps; (**B**) Number of total faults; (**C**) Total faults/total steps ratio; (**D**) Number of forelimb faults (*: *p* < 0.05).

Among the motor coordination tests, one of the most reliable indicators in our previous studies has been the grid walking/foot fault test [20,23,25]. On counting the number of steps, we found that the enriched rats moved more than the control group on the 3rd week, while there was no difference on the 4th week (Figure 4A). In the foot-fault test the control MSG-treated rats made more mistakes than the control saline-treated animals on week 4, but this difference could not be observed between the groups of the enriched control or enriched MSG-treated rats (Figure 4B). Because rats take more steps with their forelimbs than with their hindlimbs on the grid, we counted the number of forelimb foot-faults separately, which resulted in statistically significant differences (Figure 4D). The ratio of total faults compared to the number of total steps also resulted in significant differences between control and enriched groups: only the control MSG-treated rats had a significantly increased fault/total step ratio (Figure 4C). These above-mentioned differences were mainly observed on week 4, while on week 3, the differences were only observed between control and enriched groups. These data show that the development of motor coordination required for walking on a grid is delayed in MSG-treated animals, but this delay can be prevented by environmental enrichment.

**Figure 5:**
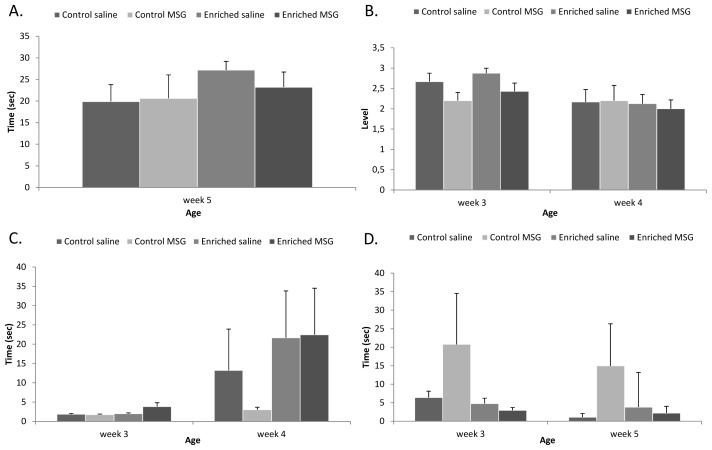
Motor coordination tests II. (**A**) Rope suspension test; (**B**) Inclined board test; (**C**) Rota-rod test; (**D**) Walk initiation test—inner circle.

In rope suspension and inclined board tests we found that pups in enriched environment performed slightly better than control animals, but differences were not significant (Figure 5A,B). There was no difference between any of the groups in the rota-rod test on week 3: all animals fell of the rotating rod in a few seconds. By week 4, animals showed significant improvement and could stay on the rod for longer times. Control MSG-treated rats, however, did not show this improvement, and could stay on the rotating wheel for a short time only, but they improved when growing up in an enriched environment (Figure 5C). Finally, walk initiation test from inner circle showed that control MSG-treated rats took markedly longer times to move out of the inner circle on both weeks 3 and 5 (Figure 5D).

### 2.4. Discussion

Our results show that while raising pups in enriched environment does not considerably alter neurobehavioral development, it can decrease the delaying effects of the excitotoxic MSG treatment.

One of the most marked effects on the physical development was the pronounced less weight gain in MSG-treated pups. This was completely abolished in pups kept in enriched cages. Some studies have reported decreased weight gain in rats raised in enriched environment [26], but we did not find significant differences in control enriched animals. The finding that enrichment could counteract the effect of MSG in weight gain must therefore be due to the action of enriched environment against the neurotoxic effects of MSG.

MSG treatment in neonatal rats has been described to lead to various biochemical, endocrinological and behavioral alterations [27–30]. MSG-treated rats are known to have slower weight gain in the beginning followed by increased weight gain in adulthood. In the central nervous system, the most pronounced excitotoxic degeneration can be observed in the arcuate nucleus and the retina, but neuronal loss and morphological changes have been described also in the cortex and hippocampus [18–22]. Considering behavioral parameters, alterations in novelty seeking [21], decreased habituation rate and locomotion [31], and changes in exploratory behavior have been described [29]. We have previously described that neonatal MSG treatment causes neurobehavioral delay as shown by delayed reflex development, slower maturation of motor coordination and disturbed novelty seeking behavior [20,21]. Several protective strategies have been used in MSG-induced lesions, including neurotrophic growth factors such as pituitary adenylate cyclase activating polypeptide [7], urocortin [32] and diazoxid [33]. Previously we have shown that enriched environment is protective in MSG-induced retinal degeneration [6]. In the present study we have provided evidence that the effects of environmental enrichment are more general: it can decrease the delayed neurobehavioral development in MSG-treated pups. However, not all effects are counteracted by enriched environment. Although the reason for these differences is not known, we have observed similar selective protective effects with other methods [34].

Enriched environment is a popular protective strategy in various neuropathological conditions. Several behavioral parameters are altered in animals raised under enriched conditions, including less stereotypic repetitive movements [35], decreased memory decline in aging [36], less depressive-like symptoms [37], attenuated response to phsychostimulants [38] and effects on risk-taking behavior [39]. Advantageous effects of enriched environment have been described in traumatic, ischemic and toxic neuronal lesions [9,40]. Enriched environment reduces both functional deficits and morphological lesions in various types of injuries, such as 6-OHDA-induced striatal lesion [41], cortical impact-induced traumatic brain injury [42] or neonatal hypoxic-ischemic injury [43]. Sensory functions have also been described to be ameliorated upon exposure to enriched environment, including visual performance and protection in retinal degeneration [6,44]. However, not all experimental data reveal only positive and protective effects of enriched environment. Among others, the neurotoxic and rewarding effects of methamphetamine could not be reduced by enrichment [45], or neurobehavioral deficits were not ameliorated by enrichment in apolipoprotein-E deficient mice [46]. Therefore, it is important to outline the types of lesions where enriched environment can bring about positive effects. In MSG-induced toxic lesion of the retina, we have described that keeping newborn rats in larger and enriched cages can markedly reduce the degeneration in the retina caused by the excitotoxic lesion [6]. Fisher and coworkers have described that MSG-induced learning deficit can be reduced by exposure to enriched housing conditions [47]. Considering postnatal behavior, the adverse effects of maternal separation on behavioral parameters have been described to be compensated by enriched rearing conditions [48], which is in accordance with our present findings in a different (toxic) adverse condition.

## 3. Experimental Section

### 3.1. Experimental Animals

A local Wistar rat colony was used for our experiments. Animal housing, care and application of experimental procedures were in accordance with institutional guidelines under approved protocols (No: BA02/2000-15024/2011, University of Pecs following the European Community Council Directive). Animals of both sexes were cross-fostered immediately after birth, to minimize litter differences. Litter size was 8 ± 1 pups in all groups. Pups stayed with their mothers during the whole examination period and were weaned after 5 weeks of age. All experimental animals were kept in the same room, under the same illumination and other outside environmental conditions (12 h light-dark cycle, food and water ad libitum).

### 3.2. Environmental Enrichment

Pups were placed in one of the following two cages immediately after birth, similarly to our earlier descriptions [6]. (1): Normal control rats were placed in a regular (control) cage with 43 × 30 × 20 cm^3^ dimensions (*n* = 30). Control rats were only shortly handled, for the duration of the neurobehavioral testing; (2): A second group of pups (*n* = 37) was placed in a large cage, the floor of which was 88 × 50 cm^2^ with 44 cm high walls (88 × 50 × 44 m^3^) supplemented with a complex environmental enrichment. Rats were continuously exposed to intensive multisensory stimulation. The cage contained different toys, objects, running tunnels and rotating rods with various shapes, materials (wood, plastic, metal), colors and shades. Half of the objects were changed daily, while the other half were left unchanged to avoid a stressful change of the environment.

### 3.3. MSG Treatment

Half of the pups in each cage (control group: *n* = 14, enriched group: *n* = 20) were injected subcutaneously with 4 mg/g bodyweight MSG (Sigma, Budapest, Hungary) dissolved in 100 μL physiological saline on postnatal days 1, 5 and 9, according to previous descriptions [49]. The other half of the litters (control group: *n* = 16, enriched group: *n* = 17) received the same volume of physiological saline.

### 3.4. Examination of Neurobehavioral Development

Examinations of neurobehavioral development were started on the first postnatal day (PND) and were carried out daily between 12 and 15 p.m. until PND 21. Neurobehavioral testing was performed in a blinded fashion, the investigator was not aware of the nature of handling. Neurobehavioral maturation and development of motor coordination were tested based on earlier descriptions [20,21,25,34,50–53].

The development of the pups was followed by many different examinations to get a wide view about the neurobehavioral development of the rats. Bodyweight was measured daily until 3 weeks of age, then twice a week until 5 weeks of age. Physical development was followed by inspections of maturation of physical characteristics such as eye opening, incisor eruption and ear unfolding. Pups were also tested for the following neurological signs and reflexes: (1) Surface righting reflex: rats were placed in supine position and the time was recorded in seconds to turn over to prone position and place all four paws in contact with the surface. (2) Negative geotaxis: animals were placed head down on an inclined grid (45°) of 30 cm. The hindlimbs of the pups were placed in the middle of the grid. The day they began to turn around and climb up the board with their forelimbs reaching the upper rim was observed. In cases where the animal did not turn around and climb up the board within the observed 30 s, the test was considered negative. From the day of the appearance of the negative geotaxis, the time in seconds to reach the upper end of the board was recorded daily. (3) Crossed extensor reflex: the left rear paw was pinched and the animal was observed for the extension of the right leg. The day of disappearance of the crossed extensor reflex in its pure form, when it was replaced by a more complex behavioral response, was noted. (4) Sensory reflexes: the ear and the eyelid were gently touched with a cotton swab and the first day of the ear twitch reflex and the contraction of the eyelid were recorded. (5) Limb placing: the back of the forepaw and hindpaw was touched with the edge of the bench while the animal suspended, and the first day of lifting and placing the paws on the table was noted. (6) Limb grasp: the fore- and hindlimbs were touched with a thin rod, the first day of grasping onto the rod was recorded. (7) *Gait*: the animals were placed in the center of a white paper with a circle of 13 cm in diameter, the day the pup began to move off the circle with both forelimbs was recorded. In cases the animal did not leave the circle for 30 s, the test was considered to be negative. From the day of the appearance, the time in seconds to move off the circle was recorded daily. (8) Auditory startle: the first day of the startle response to a clapping sound was observed. (9) Air righting: subjects were dropped head down onto a bed of shavings from a height of 50 cm, and the day of first landing on four feet was recorded.

### 3.5. Motor Coordination Tests

Rat pups were tested for motor coordination twice a week between 2 and 5 weeks of age. (1) Grid-walking and footfault test: rats were placed on a stainless steel grid floor (20 × 40 cm^2^ with a mesh size of 4 cm^2^) elevated 1 m above the floor. For a 1-min observation period, the total number of steps was counted (calculated by total right and left forelimb steps). The number of footfault, when the animals misplaced a forelimb or hindlimb that it fell through the grid, was also recorded during a 1-min period (mistakes of all four limbs were counted separately during the examination). (2) Walking initiation: rats were placed on a horizontal surface in the center of two concentric circles with diameters of 10 and 45 cm (inner and outer circles). The time taken to move off the circles was recorded. (3) Rope suspension test: animals were suspended by both their forepaws on a horizontal, 4-mm-diameter nylon rope, stretched horizontally 40 cm over a foam pad. The time the animals could hang on the rope was recorded (maximum 30 s). (4) Inclined board test: rats were placed on a wooden board, and the board was gradually elevated by 5 degrees. The maximum angle at which the animals could maintain position on the inclined board for 5 s was recorded [graded from 1 (lowest angle) to 5 (highest angle)]. (5) Rota-rod test: animals were tested on a commercially available treadmill with diameter of 14 cm for small animals, attached to a rotating motor. The test was performed at a speed of 13 rpm. The pups were placed on the rotating drum and the time the animal could stay on the rota-rod was measured (maximum 2 min).

### 3.6. Statistical Analysis

Data are expressed as mean ± standard error of the mean (SEM). Statistical analysis was performed using two-way analysis of variance (ANOVA) followed by Bonferroni-Dunn’s posthoc analysis. Results were considered significant when *p* < 0.05. Results are represented with * in case of significance level 0.01 < *p* < 0.05; ** when 0.001 < *p* < 0.01 and *** when *p* < 0.001.

## 4. Conclusions

In summary, our present results show that enriched housing conditions can reduce the delaying effects of the excitotoxic glutamate treatment on neurobehavioral development in newborn rat pups.
